# Tobacco smoke induced hepatic cancer stem cell-like properties through IL-33/p38 pathway

**DOI:** 10.1186/s13046-019-1052-z

**Published:** 2019-01-28

**Authors:** Chunfeng Xie, Jianyun Zhu, Xueqi Wang, Jiaqi Chen, Shanshan Geng, Jieshu Wu, Caiyun Zhong, Xiaoting Li

**Affiliations:** 1 Department of Toxicology and Nutritional Science, School of Public Health, Nanjing Medical University, 101 Longmian Ave, Jiangning, Nanjing, 211166 Jiangsu China; 2 Suzhou Digestive Diseases and Nutrition Research Center, North District of Suzhou Municipal Hospital, The Affiliated Suzhou Hospital of Nanjing Medical University, No. 242 Guangji Road, Suzhou, 215008 Jiangsu China; 3 Collaborative Innovation Center for Personalized Cancer Medicine, Center for Global Health, School of Public Health, Nanjing Medical University, Nanjing, 211166 China

**Keywords:** Tobacco smoke, Liver CSCs, IL-33, p38 MAPK, EMT

## Abstract

**Background:**

Tobacco smoke (TS) critically contributes to the development of hepatocellular carcinoma. Cancer stem cells (CSCs) induced by TS is an early event in the initiation of carcinogenesis. Tumor specific microenvironment including inflammatory factors is key mediator for maintaining the stemness of CSCs through various pathways such as p38 MAPK. However, the mechanisms of inflammatory factors in TS-induced acquisition of liver CSCs properties remain undefined. The aim of this study was to investigate the role of IL-33/p38 axis in long term TS-induced acquisition of hepatic CSCs properties in mouse liver tissues and human liver cells.

**Methods:**

BALB/c mice were exposed to TS for 12 weeks, along with or without 1 mg/kg SB203580 (p38 inhibitors) treatment. Histopathological analysis, alterations in the levels of IL-33, liver CSCs markers, EMT-like changes and p38 MAPK activation in liver tissues of mice were analyzed by immunohistochemical staining, immunofluorescence assay and Western blot analysis. Moreover, LO2 immortalized human liver cells were exposed to cigarette smoke extract (CSE) and the tumorsphere formation ability was determined. LO2 cells were further treated with IL-33 or CSE and the expression of phosphorylated p38, liver CSCs markers and EMT-related proteins was examined.

**Results:**

Long term TS exposure increased the levels of CSCs markers, induced epithelial-to mesenchymal transition (EMT) and inflammatory factor IL-33 expression. Moreover, we showed that p38 MAPK modulated TS-stimulated hepatic CSC-like properties, as evidenced by the findings that long term TS exposure activated p38, and that TS-induced stemness was abolished by p38 inhibition. In addition, data from in vitro model showed that similar to cigarette smoke extract (CSE), IL-33 treatment promoted the activation of p38, increased the levels of liver CSCs markers expression and EMT-like changes.

**Conclusions:**

Collectively, these data suggested that IL-33/p38 axis plays an important role in long term TS exposure-induced acquisition of hepatic CSC-like properties.

## Introduction

Tobacco smoking (TS) is an important contributor of hepatocellular carcinoma (HCC). Epidemiological studies have reported a significant positive correlation and a dose-response relationship between TS and risk of hepatocellular carcinoma [1, 2]. TS induces extensive histopathological changes, mainly parenchymatous degeneration, in the liver of neonatal mice [3, 4]. Postnatal exposure of mice to ABP, a trace component of cigarette smoke, results in a higher incidence of liver tumors [5]. Overwhelming evidences have demonstrated that TS promotes various cancer biological processes including cell migration, invasion, immune modulation as well as cancer stem cells (CSCs).

CSCs hypothesis postulates that cancer originates from the malignant transformation of stem/progenitor cells which exhibit the capability of tumor initiation. Accumulating evidences support the existence of hepatic cancer stem cells (hCSCs) in both mouse and human liver cancer tissues [6, 7]. It has been showed that hCSCs express specific cell-surface markers and critical stemness regulators, including EpCAM, CD133 [7, 8], Nanog and Oct4 [9, 10]. In addition, various studies have shown that tumor microenvironment comprising stromal cells, immune cells, networks of cytokines and growth factors are responsible for maintaining the stemness of hCSCs.

Inflammation plays an important role in tumor specific microenvironment which is thought to facilitate all phases of tumorigenesis, from initiation to metastasis [11]. Inflammatory mediators exhibit the potential to induce the acquisition of CSCs properties [12, 13]. TS exposure leads to the release of pro-inflammatory cytokines such as Interleukin-33 (IL-33) [14, 15]. IL-33, a member of the IL-1 family, has been implicated in the progression of various cancers [16]. Fang M et al. demonstrated that IL-33 stimulated colon cancer cell sphere formation and activated CSCs genes [17]. IL-33 promoted EMT process of human kidney (HK)-2 cells [18]. Numerous studies have elucidated that IL-33 is strongly associated with hepatic inflammation [19, 20]. Nevertheless, the effect of IL-33 on liver CSCs properties induced by TS is still unknown.

IL-33 regulates liver inflammation through the activation of p38 mitogen-activated protein kinases (p38 MAPK). Phosphorylation of p38 is able to transduce various signals and elicit a wide range of cellular response [21]. Suppression of p38 activity reduced the expression of CSCs markers and sphere formation ability, and decreased migratory potential in head and neck squamous cell carcinoma [22]. Previous study showed that TS triggered MAPK pathways activation in the liver of BALB/c mice [23]. However, it remains unclear whether TS induces CSCs properties of liver cells through IL-33/p38 axis.

CSCs exhibit biological traits of the epithelial-to mesenchymal transition (EMT) [24, 25]. EMT is involved in various pathological processes including tumor development [26, 27]. Our previous study revealed that TS-induced liver EMT was associated with MAPK pathway activation in the liver of mice [23]. However, whether TS-triggered CSC properties are related to IL-33/p38-mediated hepatic EMT remains to be determined. Therefore, the present study aimed to investigate the role of IL-33/p38 axis in long term TS exposure-induced acquisition of hepatic CSC-like properties.

## Methods

### Mice and tobacco smoke exposure

The protocol of mice exposed to TS has been reported in our previous study [23]. Briefly, male BALB/c mice weighing 18–22 g (8-week-old) were purchased from the Animal Research Center of Nanjing Medical University. Mice were handled under the recommendations in the guidelines of the Animal Care and Welfare Committee of Nanjing Medical University (IACUC-14030181). Ten mice were randomly assigned to each group. Mice in the control group were exposed to filtered air, tobacco smoke exposure group were exposed to tobacco smoke in a smoking apparatus. Smoke was delivered to the whole-body exposure chambers with a target concentration of total particulate matter (TPM) of 85 mg/m^3^. Mice were exposed to tobacco smoke for 6 h daily for 12 consecutive weeks. After the last tobacco smoke exposure, mice were sacrificed, and the liver tissues were isolated and stored for analysis.

### p38 MAPK inhibitor treatment of mice

Mice were treated with SB203580 (p38 inhibitors) as previously reported in a separate set of animal study. Prior to feeding, SB203580 was dissolved with corn oil. Animals were randomly divided into four groups (*n* = 8 per group): filtered air group, mice were exposed to filtered air and received control diet (AIN-76A) containing corn oil; tobacco smoke exposed group, mice were exposed to tobacco smoke and received control diet containing corn oil; tobacco smoke + DMSO group, mice were exposed to tobacco smoke and received control diet supplemented with DMSO; tobacco smoke + SB203580 group, mice were exposed to tobacco smoke and received control diet supplemented with SB203580 at dose of 1 mg/kg body weight/day. Mice were weighed every three days. The administration dosages of SB203580 were based on the measurements of mouse body weight and the amount of diet consumption. Mice were exposed to filtered air or tobacco smoke 6 h daily for 12 weeks. After exposure, mice were sacrificed and the liver tissues were collected and stored for histopathological examination, immunohistochemical staining, immunofluorescence assay, and Western blot analysis.

### Tissue immunohistochemical staining

Immunohistochemical staining was performed using the Vectastain Elite ABC Kit (Vector Laboratories, Burlingame, CA, USA) according to the manufacturer’s instructions. Briefly, paraffin-embedded sections were deparaffinized and hydrated in xylene, ethanol and water. After heat-induced antigen retrieval procedure, sections were incubated overnight at 4 °C with primary antibody, including EpCAM (21050–1-AP), CD133 (18470–1-AP), Nanog (14295–1-AP), Oct4 (60242–1-Ig), IL-33 (12372–1-AP), E-cadherin (20874–1-AP), ZO-1 (21773–1-AP), Vimentin (10366–1-AP) and N-cadherin (22018–1-AP). The above primary antibodies were purchased from Proteintech (Rosemont, IL, USA). After the primary antibody was washed off, the ABC detection system was performed by using biotinylated anti-rabbit IgG or biotinylated anti-mouse IgG. The slides were counterstained with haematoxylin and mounted in xylene mounting medium for examination. With H&E staining, 1.5 mm-in diameter tissue cores were punched from the selected tissue areas and placed on a recipient block. The quantification of immunohistochemical staining was measured with the integral optical density (IOD) ratio of positive areas relative to the total tissue areas by Image-Pro-Plus 6.0 software. Eight mice in each group and three liver slices for each animal were analyzed.

### Cell culture and cigarette smoke extract preparation

Human immortalized liver LO2 cells obtained from Shanghai Institute of Cell Biology, Chinese Academy of Sciences (Shanghai, China) were cultured in RPMI 1640 medium supplemented with 100 U/mL penicillin, and 100 μg/mL streptomycin (Gibco, Gaithersburg, MD) and incubated in 5% CO_2_ at 37 °C. Cell layers were 70–80% confluent at the time of cigarette smoke extract (CSE) exposure. CSE was prepared daily immediately before use according to the reported method [28]. Briefly, one filterless 3R4F Research Reference Cigarette (University of Kentucky, 9 mg tar and 0.76 mg nicotine/cigarette) was combusted and the mainstream smoke was continuously drawn through a glass syringe containing 10 ml of fetal bovine serum free RPMI 1640 medium that was pre-warmed to 37 °C at a rate of 5 min/cigarette. The resulting suspension was adjusted to pH 7.4 and then filtered through a 0.22-μm-pore size filter. The obtained solution was referred to as a 100% CSE solution and further diluted to 1% CSE with culture medium. Control solution was prepared with the same protocol, except that the cigarette was unlit. LO2 cells were exposed to CSE within 30 min of preparation.

### Immunofluorescence assay

Tissues and cells were fixed with 4% paraformaldehyde and permeabilized with 0.1% Triton X-100. Phosphorylated-p38 antibody was applied at 4 °C overnight. FITC-conjugated goat anti-mouse IgG was used as secondary antibody. Fluorescently-labeled secondary antibody was added and incubated for 1 h at room temperature. Eventually, nuclei were counterstained with DAPI. The stained tissues and cells were mounted on glass slides and examined by confocal microscopy.

### Western blot analysis

Liver tissues and cells were harvested after indicated treatments and lysed using RIPA buffer containing 1x protease inhibitor cocktail (Pierce) and EDTA. Bradford Protein Assay Kit was used to quantify the amount of protein in the total protein lysates. Equal amounts of a total of proteins (60 μg) were separated on 10% SDS-PAGE and transferred to PVDF membranes (Bio-Rad) for immunoblotting. The membranes were blocked with 5% non-fat dry milk at room temperature for 1 h on a rotary shaker, followed by overnight incubation at 4 °C with primary antibodies. The membranes were washed with Tris-buffered saline/Tween (TBS/T) and then incubated with the secondary antibody. The following primary antibodies were used: EpCAM, CD133, Nanog, Oct4, IL-33, E-cadherin, ZO-1, N-cadherin, Vimentin, p38 (14064–1-AP) (Proteintech, Rosemont, IL, USA); phosphorylated p38 (# 4511), c-Jun (# 9165), phosphorylated c-Jun (# 2361), JunD (# 5000), c-Fos (# 2250), phosphorylated c-Fos (#5348), and FosB (# 2236) (Cell Signaling Technology, Beverly, MA, USA). β-actin (AP0060) was purchased from Biogot Technology (Nanjing, China).

For densitometric analyses, protein bands on the blots were measured after normalization to β-actin with Eagle Eye II software. To estimate the increase of p38, c-Jun and c-Fos phosphorylation, a ratio between their phosphorylated form and the relative total protein was determined.

### Tumorsphere formation assay

LO2 cells were exposed to 0% or 1% concentrations of CSE for 14 days and then cultured in 6-well ultralow attachment plates with a serum-free tumorsphere culture medium (DMEM/F12) (Gibco) for another 7 days. The tumorsphere culture medium was supplemented with 4 μg/mL insulin, 10 ng/mL basic fibroblast growth factor (bFGF), and 20 ng/mL human recombinant epidermal growth factor (EGF). The images of representative fields were acquired and the numbers of tumorspheres were counted under a microscope (sphere diameter > 50 μm was counted).

### Statistical analysis

Statistical analyses were performed with SPSS 25.0. All data were expressed as mean ± standard deviation. One-way ANOVA was used for comparison of statistical differences among multiple groups. Unpaired two-tailed Student’s *t* test was also used for the comparison between two groups. A *P* value of < 0.05 was considered significantly different.

## Results

### Long term TS exposure induced liver cells acquisition of CSC-like properties

**Fig. 1:**
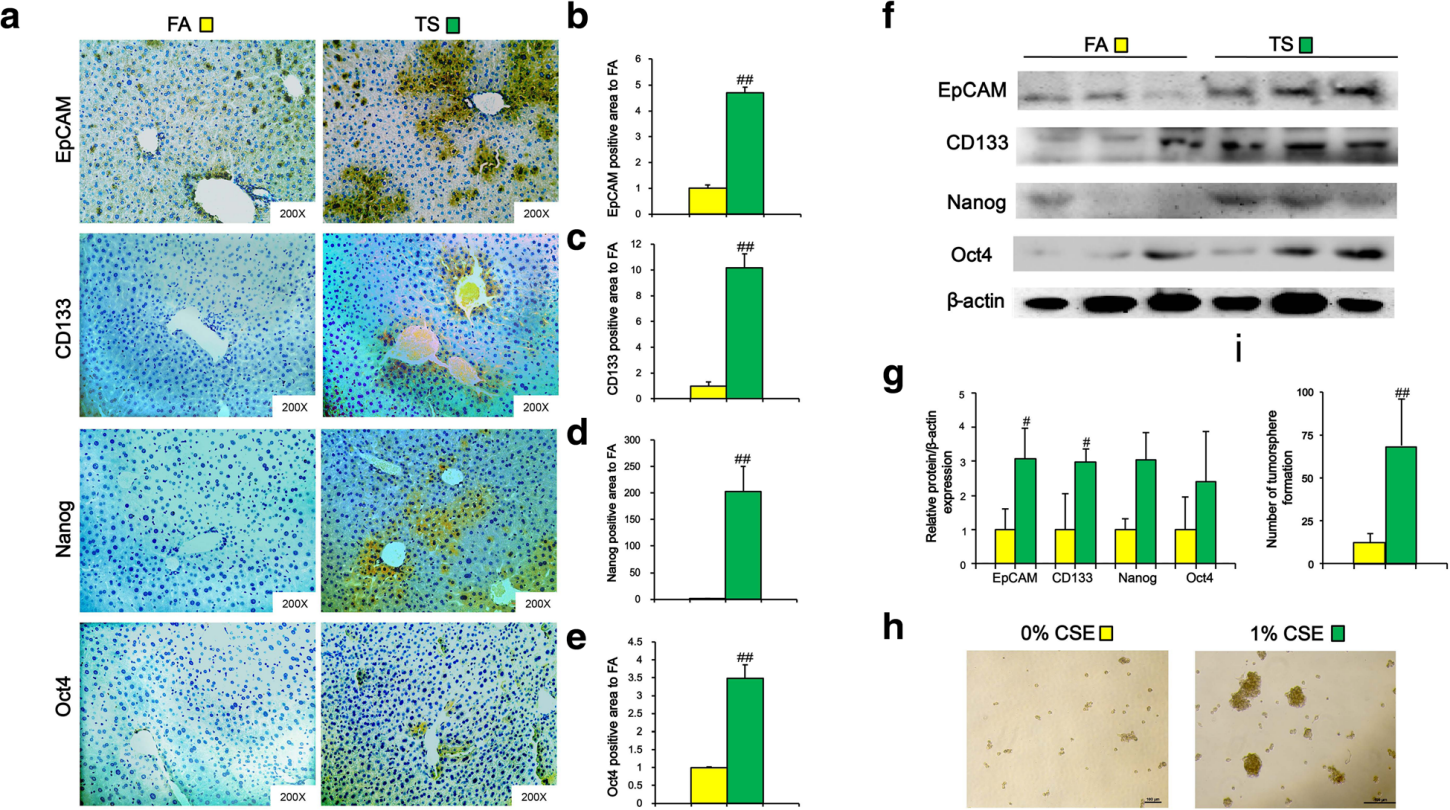
Long term TS exposure induced liver cells acquisition of CSC-like properties. Mice were exposed to TS for 12 weeks. The alternation of liver CSCs markers was analyzed by immunohistochemical staining (IHC) (**a**). EpCAM (**b**), CD133 (**c**), Nanog (**d**) and Oct4 (**e**)-positive areas in TS group relative to FA group. **f** Western blotting was used to analyze the expression of the indicated genes. **g** Densitometry results were shown as fold change compared with FA group after normalization to β-actin. Six animal samples per group were used for the densitometric analysis. **h-i** LO2 immortalized human liver cells were exposed to 0% or 1% concentrations of cigarette smoke extract (CSE) for 14 days and then cultured with serum free medium for another 7 days. Images of tumorsphere (**h**) and the numbers of tumorsphere (**i**) were examined. Data are expressed as mean ± SD. The significance was assessed with unpaired two-tailed Student’s *t* test. # *P* < 0.05, ## *P* < 0.01, compared with FA control or 0% CSE. FA = filtered air; TS = tobacco smoke

CSCs serve as seeds in different stages of tumorigenesis including initiation. To investigate the effects of TS on CSCs properties, mice were exposed to TS for 12 weeks and the expression of hepatic CSCs markers in liver tissues were examined by immunohistochemical staining (IHC). As shown in Fig. 1a-e, long term TS exposure significantly increased the positive areas of EpCAM, CD133, Nanog and Oct4 staining in liver tissues of mice when compared with the control group. Western blotting also showed that long term TS exposure elevated the protein expression levels of those CSCs markers in the livers (Fig. 1f and g).

To further examine the effect of cigarette smoke extract (CSE) on the acquisition of CSCs properties, LO2 immortalized human liver cells were exposed to CSE (0, or 1%) for 14 days, and the tumorsphere formation ability was determined. It was shown that 68 ± 27 spheres were formed by LO2 cells exposed to 1% CSE. In contrast, few spheres were found in the control cells (Fig. 1h and i). Therefore, these results suggested that long-term TS exposure induced acquisition of CSC-like properties in liver cells.

### Long term TS exposure-induced CSC-like properties were associated with IL-33 upregulation

**Fig. 2:**
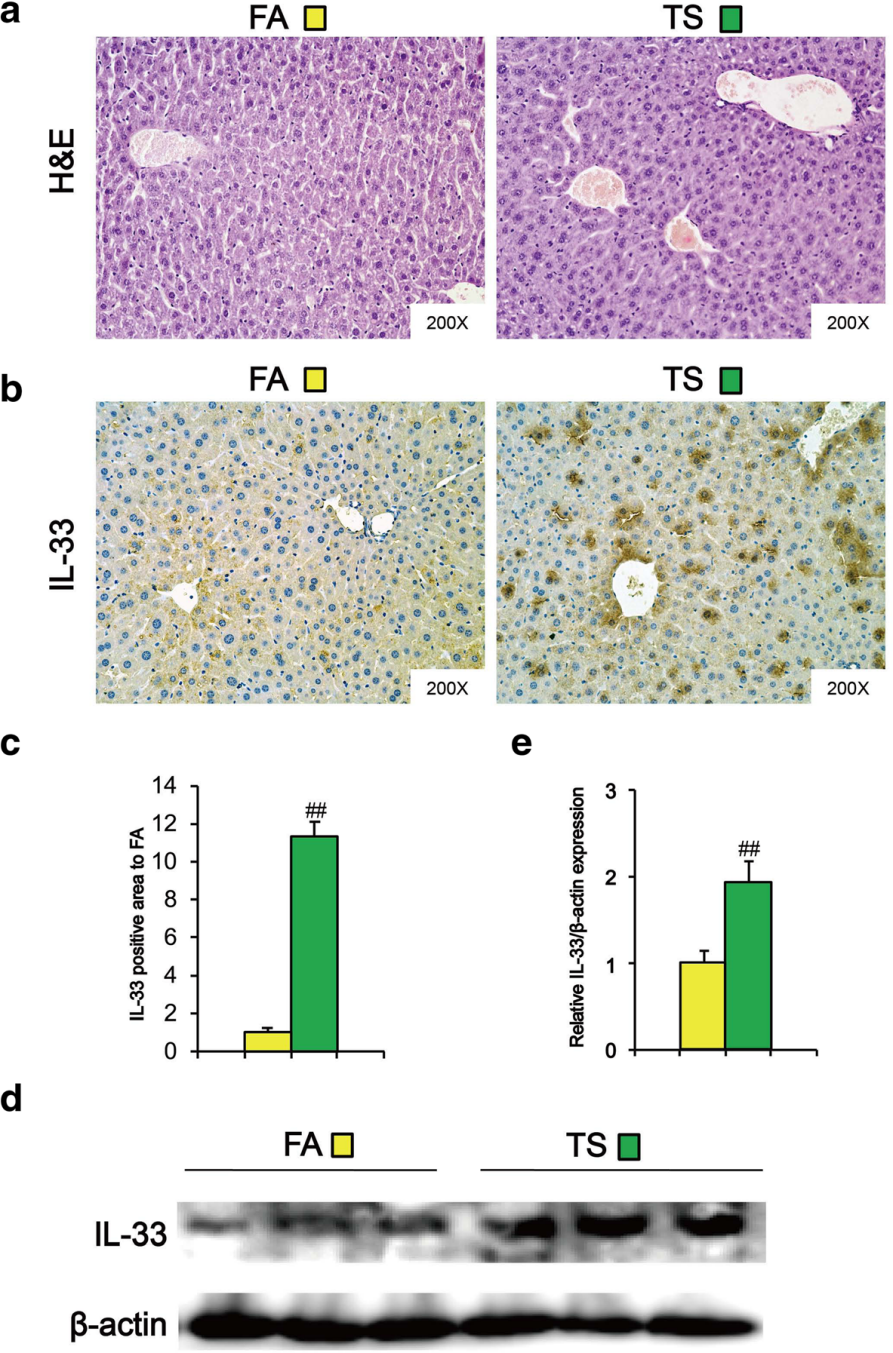
Long term TS-induced CSC-like properties were associated with IL-33 upregulation. Mice were exposed to TS for 12 weeks, and representative micrographs of liver tissues were stained with H&E (**a**). The expression of IL-33 was detected by immunohistochemical staining (**b**). **c** IL-33 positive areas in TS group relative to FA group. **d** Western blotting of IL-33 in liver tissues. β-actin was served as the loading control. **e** Six animal samples per group were used for the densitometric analysis of IL-33 protein. Data are expressed as mean ± SD. The significance was assessed with unpaired two-tailed Student’s *t* test. ## *P* < 0.01, compared with FA control. FA = filtered air; TS = tobacco smoke

To investigate if long term TS exposure promoted inflammatory response, liver tissues from different treatment groups were underwent histopathological analysis. H&E staining showed that hepatocytes in control animals exhibited normal texture without cell infiltrate, whereas TS caused demolishment of hepatocytes, as evidenced by the formation of bridging necrosis, collagen accumulation and large septa (Fig. 2a). Since IL-33 plays an important role in hepatic inflammation, we then examined the expression change of IL-33 in liver tissues following TS exposure. Immunohistochemical assay revealed that the expression of IL-33 was remarkably increased in liver tissues from TS-exposed mice in comparison with the control mice (Fig. 2b and c). Western blotting also showed the elevated level of IL-33 in liver tissues after long-term TS exposure (Fig. 2d and e). These data suggest that IL-33 upregulation was involved in the acquisition of CSC-like properties of liver tissues chronically exposed to TS.

### Long term TS exposure-induced CSC-like properties were related to p38 activation

**Fig. 3:**
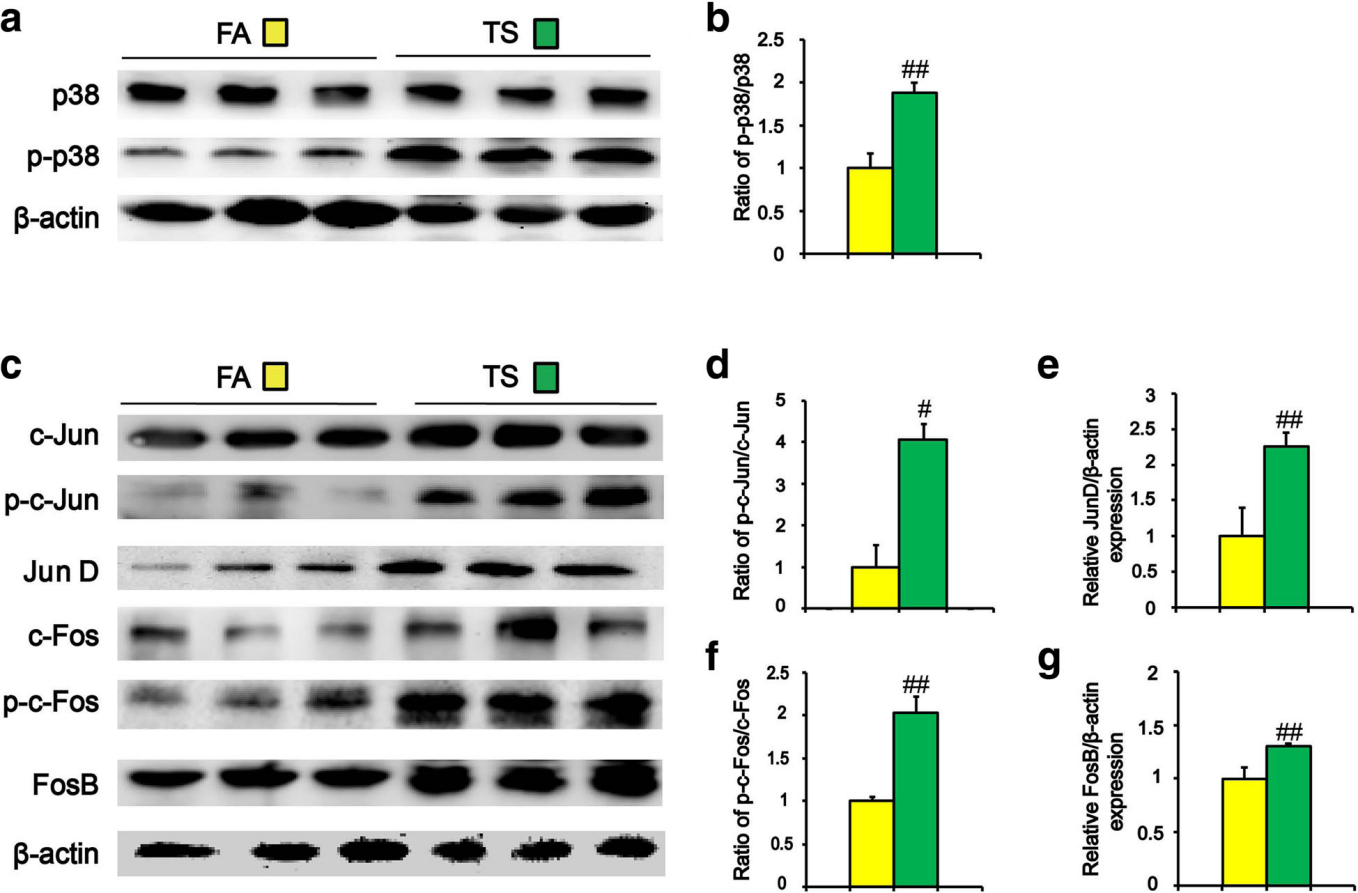
Long term TS exposure-induced CSC-like properties were related to p38 activation. Mice were exposed to TS for 12 weeks; Western blotting of p38 (**a**) and AP-1 proteins (**c**). Densitometric analyses of Western blotting for p-p-38/p-38 (**b**), p-c-Jun/c-Jun (**d**), JunD (**e**), p-c-Fos/c-Fos (**f**) and FosB (**g**) were measured after normalization to β-actin; six animal samples per group were used for the densitometric analysis. Data are expressed as mean ± SD. The significance was assessed with unpaired two-tailed Student’s *t* test. # *P* < 0.05, ## *P* < 0.01, compared with FA control. FA = filtered air; TS = tobacco smoke

Previous studies have demonstrated that IL-33 upregulates p38 activation in liver inflammatory response [18]. p38 is a critical mediator contributing to stemness acquisition and maintenance. To determine whether long term TS exposure-induced CSC-like properties correlated with the change of p38 activation, the levels of phosphorylated p-38, total p-38 as well as the ratio between p-p-38 and p-38 were measured. Compared with control group, chronic TS exposure increased level of p-p-38 and the ratio of p-p38/p38 in the liver tissues of mice (Fig. 3a and b), suggesting the promotive effect of TS on p38 activation. Meanwhile, Western blot analysis showed that TS increased the activation of AP-1 proteins in liver tissues, as indicated by increased ratios of p-c-Jun/c-Jun, p-c-Fos/c-Fos, and the levels of p-c-Jun, p-c-Fos, JunD and FosB (Fig. 3c-g).

### p38 suppression reversed long term TS exposure-triggered CSC-like properties

**Fig. 4:**
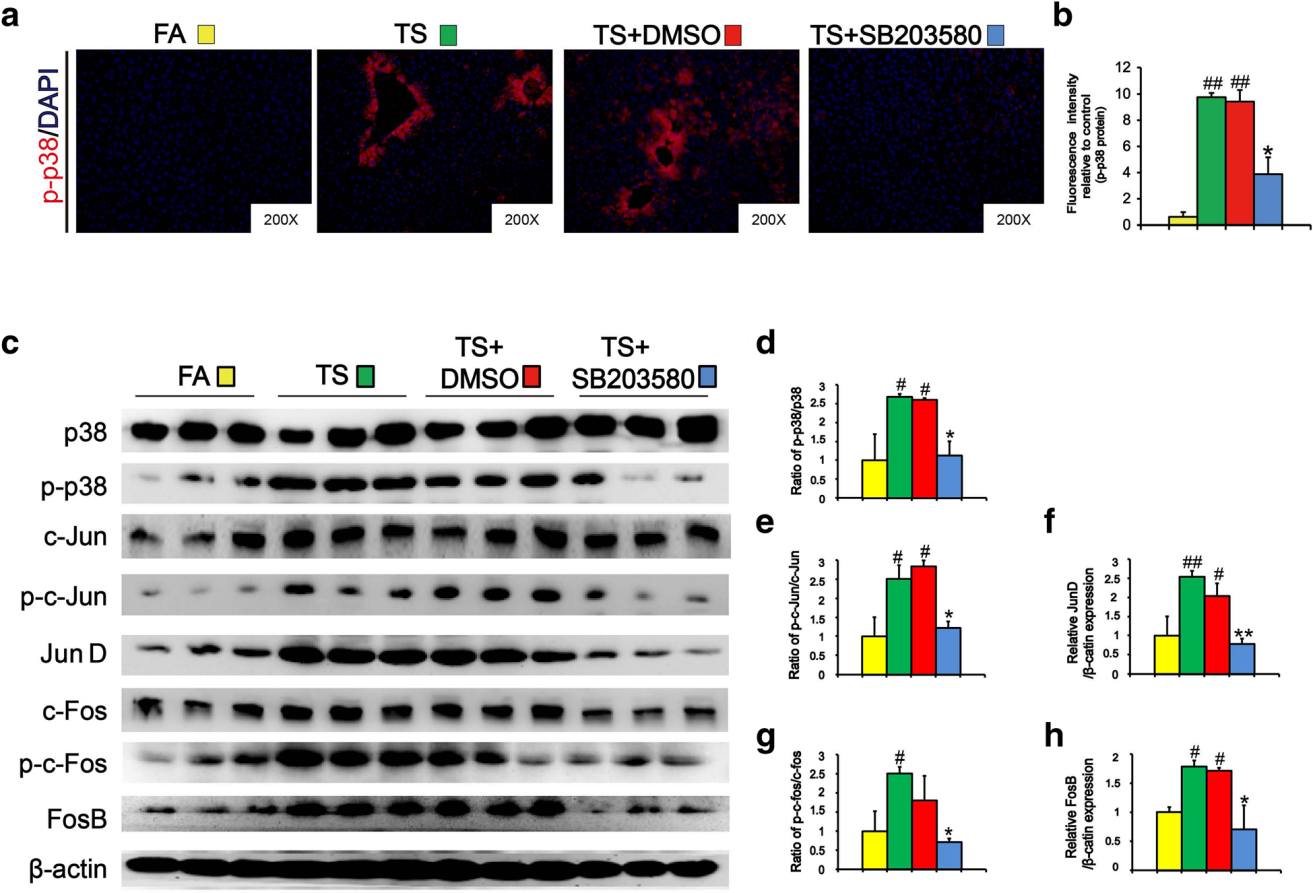
SB 203580 suppressed the activation of p38 MAPK in TS-exposed liver tissues. Mice exposed to TS were treated with or without p38 MAPK inhibitor (SB 203580) for 12 weeks, and the activation of p38 MAPK was detected by tissue immunofluorescence staining (**a**). **b** Fluorescence intensity of p-p38 relative to FA or TS + DMSO group. **c** The expression levels of p-p38, p38 and AP-1 proteins, including p-c-Jun, c-Jun, JunD, p-c-Fos, c-Fos, and FosB, were detected by Western blotting. **d-g** Densitometric analyses of Western blotting for p-p-38/p-38 (**d**), p-c-Jun/c-Jun (**e**), JunD (**f**), p-c-Fos/c-Fos (**g**) and FosB (**h**) were measured after normalization to β-actin; six animal samples per group were used for the densitometric analysis. Data are expressed as mean ± SD. The significance was assessed with one-way ANOVA test. # *P* < 0.05, ## *P* < 0.01, compared with FA control; * *P* < 0.05, ** *P* < 0.01, compared with TS + DMSO group. FA = filtered air; TS = tobacco smoke

As above results revealed the association between CSC-like properties and p38 activation in liver tissues after long-term TS exposure, we then explored the role of p38 activity in this process. Mice were received SB203580 (1 mg/kg body weight), a highly specific p38 MAPK inhibitor, along with being exposed to TS. Tissue immunofluorescence assay showed that TS-elicited p38 activation was remarkably suppressed by SB203580 treatment (Fig. 4a and b). Western blot analyses also revealed that SB203580 treatment diminished the upregulation of AP-1 proteins following TS exposure (Fig. 4c-h). These data suggested that SB203580 effectively reversed long term TS exposure-induced p38 activation in the liver tissues of mice.

**Fig. 5:**
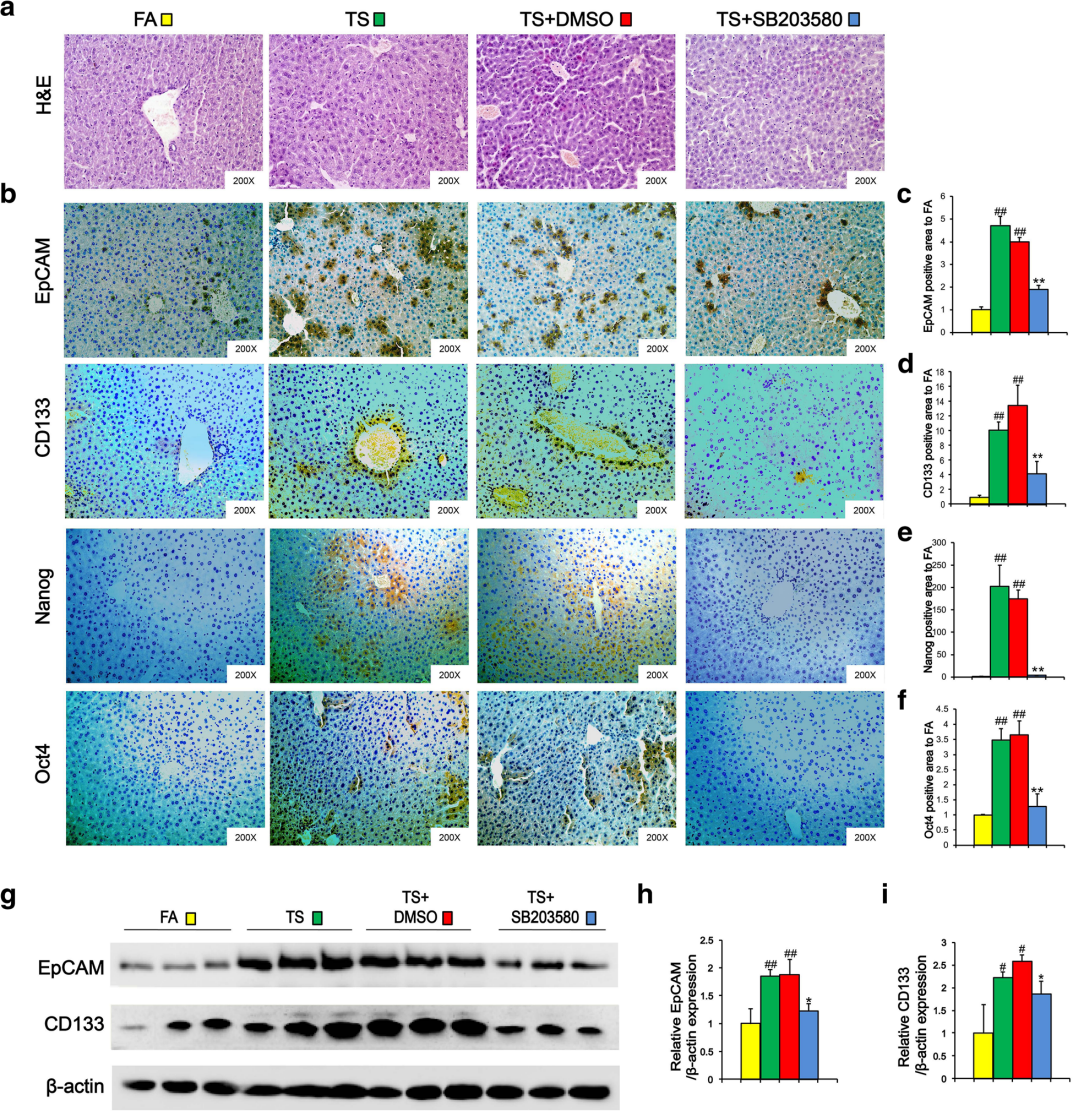
p38 suppression reversed long term TS exposure-triggered CSC-like properties. Mice exposed to TS were treated with or without p38 MAPK inhibitor (SB 203580) for 12 weeks, and representative micrographs of liver tissue were stained with H&E (**a**). **b** Immunohistochemical staining for EpCAM, CD133 and Nanog, Oct4 in liver tissues. **c-f** Fold changes of EpCAM (**c**), CD133 (**d**), Nanog (**e**) and Oct4 (**f**) - positive area in TS group compared with FA group. **g** Western blotting of EpCAM and CD133 in liver tissues. β-actin was served as the loading control. **h-i** The indicated proteins relative to β-actin were assessed by densitometric analysis; six animal samples per group were used for the densitometric analysis. Data are expressed as mean ± SD. The significance was assessed with one-way ANOVA test. # *P* < 0.05, ## *P* < 0.01, compared with FA control; * *P* < 0.05, ** *P* < 0.01, compared with TS + DMSO group. FA = filtered air; TS = tobacco smoke

Next, we examined the effects of p38 suppression on histopathological changes in the livers of mice exposed to TS. H&E staining showed that SB203580 treatment attenuated long term TS exposure-induced formation of bridging necrosis, collagen accumulation and large septa (Fig. 5a). To further assess the effects of p38 inhibition on CSC-like property in liver tissues after long term TS exposure, alterations in liver CSCs markers were analyzed by immunohistochemical assay. It was shown that TS-triggered upregulations of EpCAM, CD133, Nanog and Oct4 were abrogated by SB203580 treatment. Similar results were also showed by Western blot analysis (Fig. 5g-i). These results indicated that suppression of p38 activation reversed long term TS exposure-triggered CSC-like properties.

### TS-induced and p38-dependent EMT process could contribute to the acquisition of CSC-like properties by liver cells

**Fig. 6:**
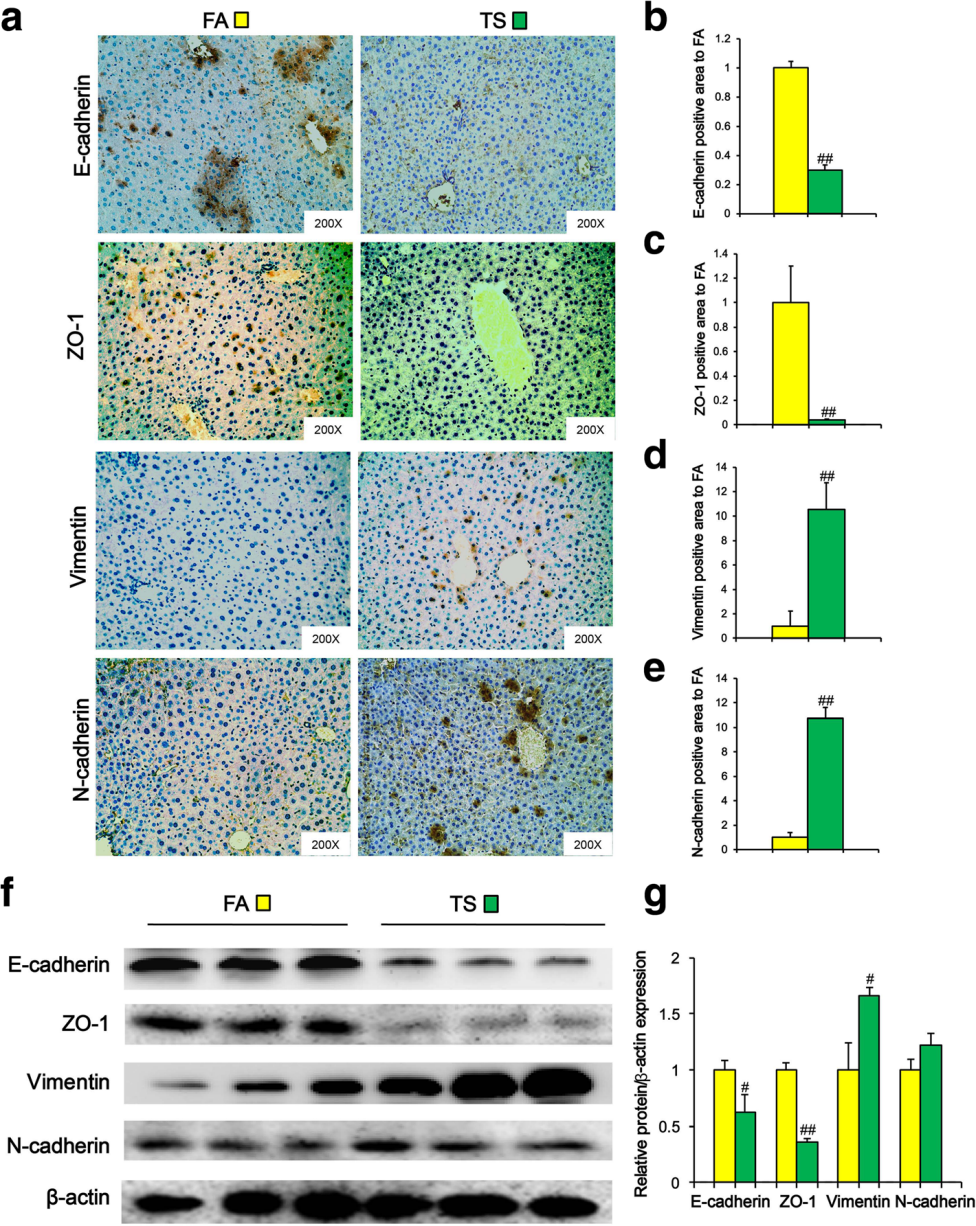
Long term TS exposure induced EMT-like alterations in liver tissues of mice. Mice were exposed to TS for 12 weeks, and the expression of E-cadherin, ZO-1, N-cadherin, and Vimentin was detected by immunohistochemical staining (**a**). **b**-**e** Fold changes of E-cadherin (**b**), ZO-1 (**c**), Vimentin (**d**) and N-cadherin (**e**)-positive area in TS group relative to FA group. (**f**) Western blotting of E-cadherin, ZO-1, Vimentin and N-cadherin in liver tissues. β-actin was served as the loading control. **g** The indicated proteins relative to β-actin were assessed by densitometric analysis; six animal samples per group were used for the densitometric analysis. Data are expressed as mean ± SD. The significance was assessed with unpaired two-tailed Student’s *t* test. # *P* < 0.05, ## *P* < 0.01, compared with FA control. FA = filtered air; TS = tobacco smoke

Emerging evidences have demonstrated that CSCs exhibit EMT potential. Previous study demonstrated that long term TS exposure induced EMT and MAPK pathway activation in the liver of BALB/c mice [23]. In line with the above report, we also found that chronic TS exposure decreased the expression of E-cadherin and ZO-1, and upregulated the expression of Vimentin and N-cadherin in liver tissues (Fig. 6a-e). These immunohistochemical results were further confirmed by Western blotting (Fig. 6f and g).

**Fig. 7:**
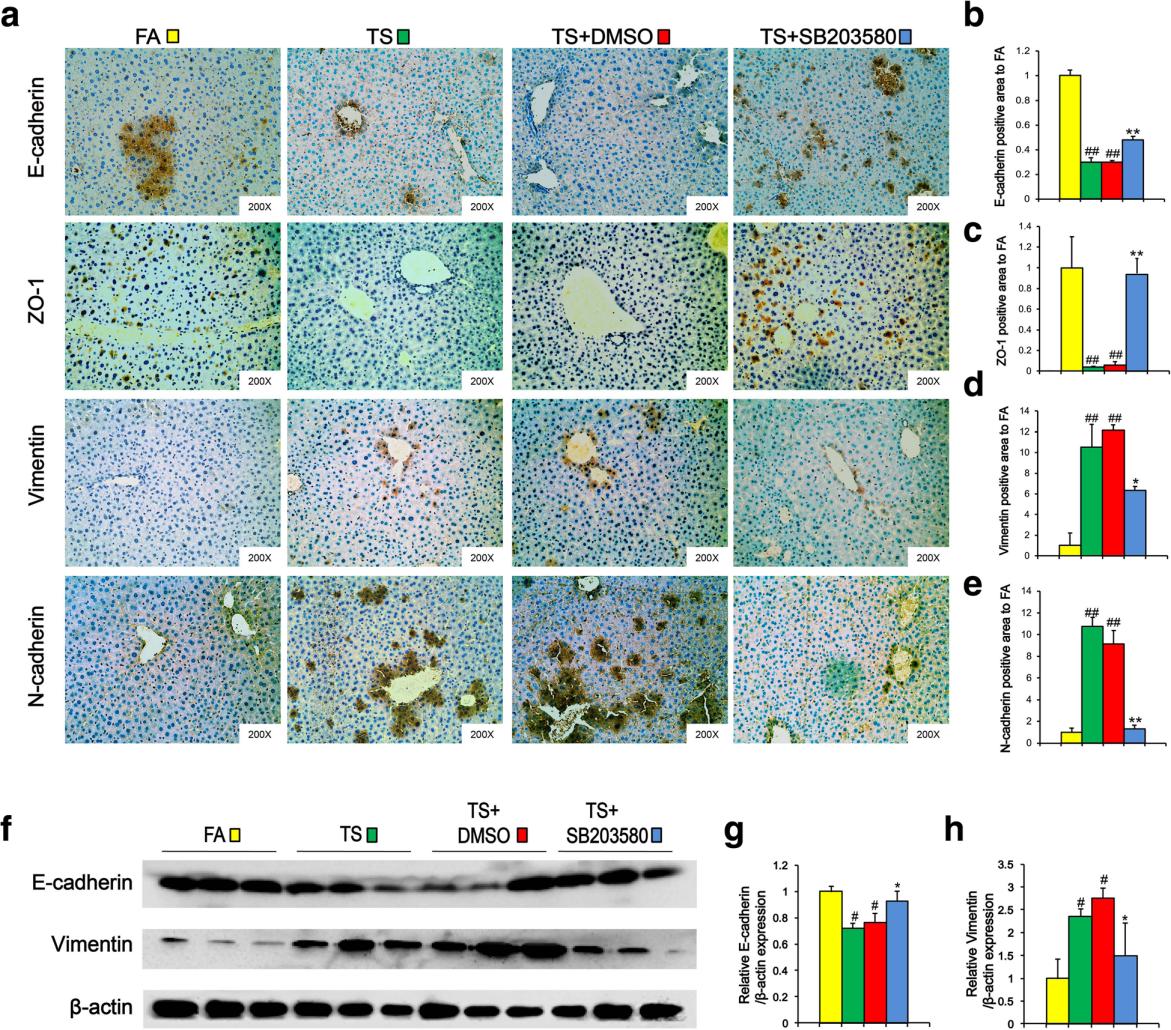
Long term TS exposure-induced EMT was involved in p-38 activation and CSC-like properties. Mice exposed to TS were treated with or without p38 MAPK inhibitor (SB 203580) for 12 weeks. **a** Liver tissues were stained immunohistochemically for E-cad, ZO-1, Vimentin and N-cad. **b-e** Fold changes of E-cadherin (**b**), ZO-1 (**c**), Vimentin (**d**) and N-cadherin (**e**)-positive area in TS group relative to FA group. **f** Western blotting of E-cadherin and Vimentin in liver tissues. β-actin was served as the loading control. **g-h** The indicated proteins relative to β-actin were assessed by densitometric analysis; six animal samples per group were used for the densitometric analysis. Data are expressed as mean ± SD. The significance was assessed with one-way ANOVA test. # *P* < 0.05, ## *P* < 0.01, compared with FA control; * *P* < 0.05, ** *P* < 0.01, compared with TS + DMSO group. FA = filtered air; TS = tobacco smoke

Moreover, we showed that TS-elicited EMT-like changes, including downregulation of E-cadherin and ZO-1, and upregulation of Vimentin and N-cadherin, were effectively abolished by p-38 suppression with SB203580 treatment (Fig. 7a-e). Similar changes were also observed by Western blotting (Fig. 7f-h). These data suggest that the TS-induced and p38-dependent EMT process could contribute to the acquisition of CSC-like properties by liver cells.

### IL-33 mimicked long term TS exposure-induced CSC-like properties in vitro

**Fig. 8:**
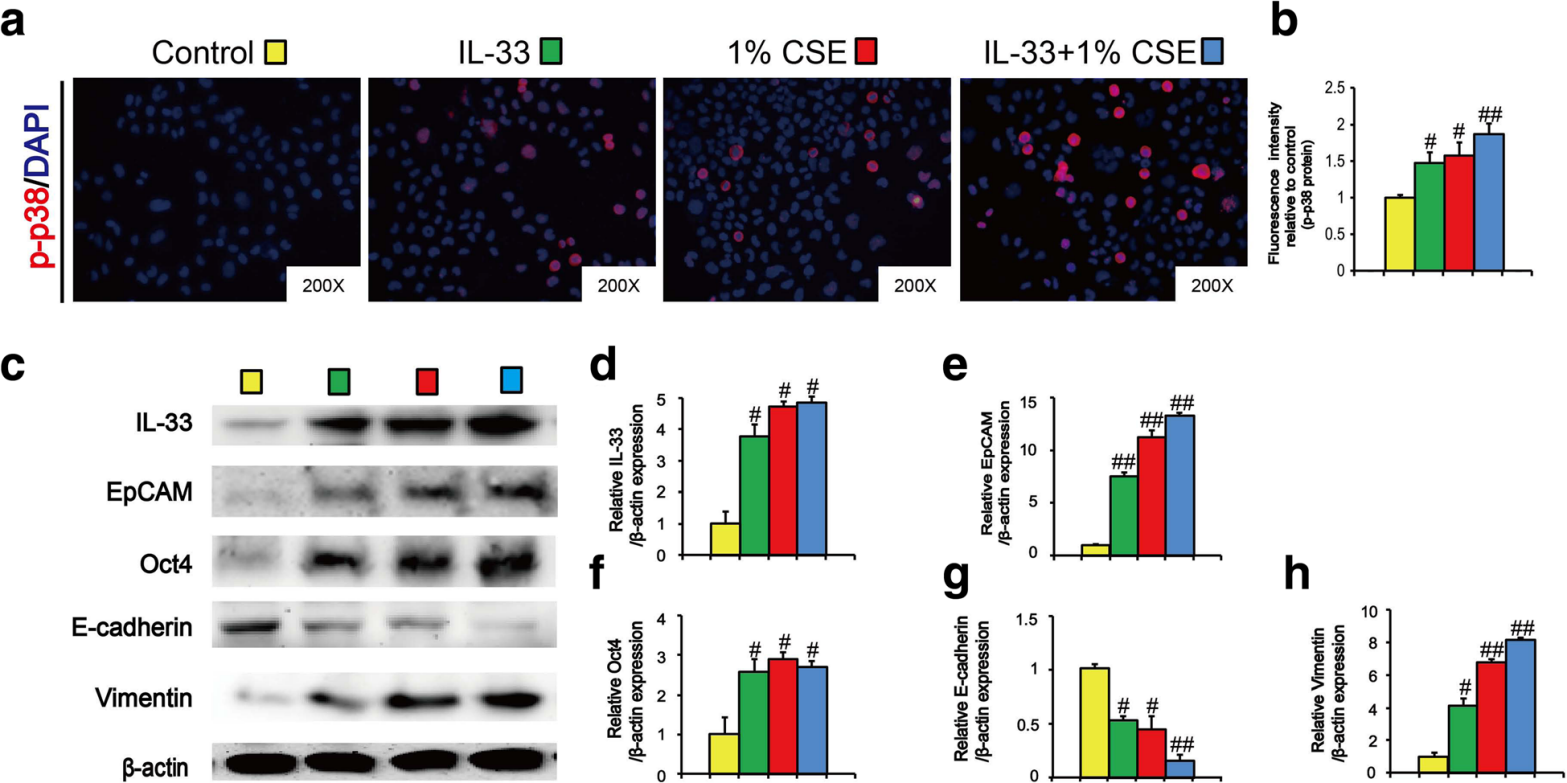
IL-33 mimicked long term TS exposure-induced CSC-like hepatic properties in vitro. LO2 cells were treated with IL-33 (10 μg/ml), CSE (1%) or IL-33 + 1% CSE for 7 days. **a** Immunofluorescent staining of p-p38 MAPK. **b** Fluorescence intensity of p-p38 relative to control group. **c** The expression levels of IL-33, EpCAM, Oct4, E-cadherin and Vimentin were detected by Western blotting. **d-h** Densitometric analyses of Western blotting for IL-33 (**d**), EpCAM (**e**), Oct4 (**f**), E-cadherin (**g**) and Vimentin (**h**) were measured after normalization to β-actin. Three independent experiments were performed. Data are expressed as mean ± SD. The significance was assessed with one-way ANOVA test. # *P* < 0.05, ## *P* < 0.01, compared with control. FA = filtered air; CSE = cigarette smoke extract

To assess whether TS-induced acquisition of hepatic CSC-like properties was associated with IL-33/p38 axis in vitro, LO2 immortalized human liver cells were treated with 1% of cigarette smoke extract (CSE) or IL-33 (10 μg/ml) for 7 days, and the expression of phosphorylated p38 was detected by immunofluorescent staining. As shown in Fig. 8a and b, IL-33 increased the fluorescence intensity of phosphorylated p38. Similar alteration was also observed in CSE-treated LO2 cells (Fig. 8a and b), suggesting that like CSE, IL-33 stimulated p38 activation in liver cells. Moreover, Western blotting results revealed that, in line with CSE treatment, IL-33 treatment induced the expression of CSCs markers (EpCAM and Oct4) as well as EMT-like changes (Fig. 8c, e-h). Collectively, these data suggested that IL-33/p38 axis has an important role in long term TS exposure-induced acquisition of hepatic CSC-like properties.

## Discussion

Tobacco smoke (TS) is an important risk of hepatocellular carcinoma. TS induction of CSCs is an early event in the initiation of carcinogenesis. Inflammation plays a critical role in maintaining the stemness of CSCs through various pathways including p38 MAPK. IL-33 exhibits a strong correlation with hepatic inflammation. However, the molecular mechanisms of IL-33 in TS exposure induced acquisition of hepatic CSCs properties remain unclear. The present study showed that long term TS exposure increased the levels of CSCs markers, induced EMT and inflammatory factor IL-33 expression in liver tissues. Moreover, we demonstrated that long term TS exposure activated p38, and that TS-induced hepatic CSC-like properties were abolished by p38 inhibition. In addition, data from in vitro model showed that similar to TS, IL-33 treatment promoted p38 activation, increased liver CSCs markers expression and EMT-like changes. Taken together, these data suggested that IL-33/p38 axis plays an important role in long term TS exposure-induced acquisition of hepatic CSC-like properties.

The stemness properties of CSCs contribute to the formation of HCC cells [29, 30]. Tumorsphere formation assay via SFM (serum-free medium) culturing has been widely used for the isolation and enrichment of CSCs in vitro. Several cell surface markers, including EpCAM and CD133, can be used to enrich liver CSCs. Studies have showed that EpCAM-positive hepatocellular carcinoma cells possess CSCs features, such as tumor initiating capacity and invasive ability [7, 31]. Ma S et al. isolated and identified a population of CD133^+^ cells from liver cancer cell lines and xenograft tumors [8]. CD133^+^ cells share stem cell properties and possess more potent abilities to form tumor in vivo [32]. Nanog is a transcription factor that is dysregulated and involved in promoting tumorigenesis through regulating CSCs characteristics [33]. Shan J et al. found that Nanog+ cells exhibited higher ability of self-renewal, clonogenicity, and initiation of tumors in human hepatocellular carcinoma [9]. The pluripotent gene Oct4 is critical in the maintenance of liver CSCs [10, 34, 35]. In the present study, we showed that long term TS exposure significantly increased the expression levels of EpCAM, CD133, Nanog and Oct4 in liver tissues of mice. Moreover, 1% CSE treatment elevated the tumorsphere formation ability of LO2 immortalized human liver cells. Together, our data suggested that TS promoted CSCs related traits in liver cells.

Accumulating evidences have implicated that IL-33 plays a critical role in tumor initiation and progression [17, 36–38]. Upregulation of IL-33 is correlated with poor prognosis in gastric cancer, non-small cell lung cancer, and hepatocellular carcinoma [36–38]. IL-33 accelerates tumor growth and metastases of breast cancer and colorectal cancer [39, 40]. Moreover, it has been showed that IL-33 regulates CSCs properties. IL-33 promotes tumorsphere formation and prevents chemotherapy-induced apoptosis of colon cancer cells [17]. IL-33 induces breast CSCs properties, as evidenced by mammosphere formation, xenograft tumorigenesis, as well as CSCs markers expression [41]. McHedlidze et al showed that chronic hepatocellular stress stimulated IL-33 release in vivo [42]. The level of IL-33 was markedly enhanced in lung tissues of mice exposed to TS [14, 43]. However, the effect of IL-33 on liver CSCs properties induced by TS remains unclear. The present study demonstrated that long term TS exposure-induced CSC-like properties were associated with remarkably elevated IL-33 in liver tissues of mice. We further showed that similar to TS, IL-33 treatment induced the expression of CSCs markers as well as EMT-like changes in human liver cells. These results suggested the involvement of IL-33 in TS -induced liver CSC-like properties.

Activation of IL-33 stimulates various signaling pathways implicating in tumor initiation, and p38 MAPK is one of the critical downstream targets of IL-33 [44, 45]. IL-33 dose-dependently induces the activity of p38 MAPK in hepatic stellate cells [20]. p38 regulates important cellular functions in response to exogenous and endogenous stimuli including TS. TS-triggered p38 activation has been detected in the bladder and gastric tissues of mice [46, 47]. Upregulation of p38 promotes CSC-like characteristics in osteosarcoma and breast cancer cells [48, 49]. p38 activity is critical for maintaining CSCs traits in head and neck squamous cell carcinoma [22]. Our previous study indicated that TS induced p38 activation in mouse liver tissues [23]. However, whether IL-33/p38 axis participates in TS-induced acquisition of liver CSCs-like properties is still unknown. In this study, we showed that long term TS exposure activated p-38 MAPK and AP-1 proteins in liver tissues of mice; inhibition of p38 MAPK abrogated TS-triggered CSC-like properties. Moreover, we showed that IL-33 treatment stimulated p38 activation and induced CSCs markers expression in LO2 immortalized human liver cells. Taken together, these data suggested the important role of IL-33/p38 axis in TS-induced hepatic CSC-like properties.

It has been demonstrated that CSCs possess EMT property and are responsible for tumor metastasis. Previous study has showed that TS triggered EMT in the liver of BALB/c mice [23]. Consistent with the above report, we found in the present study that long term TS exposure triggered EMT-like change, and that TS-induced EMT-like alteration was effectively abolished by p-38 suppression. As we described above, long term TS exposure-induced CSC-like properties were associated with IL-33 upregulation. Moreover, we showed that IL-33 triggered p-38 activation and EMT-like change in LO2 immortalized human liver cells. TS-induced and p38-dependent EMT process could contribute to the acquisition of CSC-like properties by liver cells.

## Conclusions

In summary, our data suggested that IL-33/p38 axis plays an important role in long term TS exposure-induced acquisition of hepatic CSC-like properties.

## Abbreviations

CSCs: Cancer stem cells
CSE: Cigarette smoke extract
EMT: Epithelial-mesenchymal transition
EpCAM: Epithelial cell adhesion molecule
HCC: Hepatocellular carcinoma
hCSCs: Hepatic cancer stem cells
HK-2: Human kidney-2
IHC: Immunohistochemical
IL-33: Interleukin-33
p38 MAPK: p38 mitogen-activated protein kinases
p-c-fos: phosphorylated c-fos
p-c-Jun: Phosphorylated-c-Jun
p-p38: Phosphorylated p38
SFM: Serum-free medium
TPM: Total particulate matter
TS: Tobacco smoke
