# Correction: Tobacco smoke induced hepatic cancer stem cell-like properties through IL-33/p38 pathway

**DOI:** 10.1186/s13046-023-02722-0

**Published:** 2023-06-06

**Authors:** Chunfeng Xie, Jianyun Zhu, Xueqi Wang, Jiaqi Chen, Shanshan Geng, Jieshu Wu, Caiyun Zhong, Xiaoting Li

**Affiliations:** 1 Department of Toxicology and Nutritional Science, School of Public Health, Nanjing Medical University, 101 Longmian Ave, Jiangning, Nanjing, 211166 Jiangsu China; 2 Suzhou Digestive Diseases and Nutrition Research Center, North District of Suzhou Municipal Hospital, The Affiliated Suzhou Hospital of Nanjing Medical University, No. 242 Guangji Road, Suzhou, 215008 Jiangsu China; 3 Collaborative Innovation Center for Personalized Cancer Medicine, Center for Global Health, School of Public Health, Nanjing Medical University, Nanjing, 211166 China

**Correction:** ***J Exp Clin Cancer Res*** **38, 39 (2019)**

**https://doi.org/10.1186/s13046-019-1052-z**

Following publication of the original article [1], an overlapping of images was identified Fig. 1e and Fig. 5b.

The images of Oct4 in TS and TS + DMSO groups were replaced and the correct Fig. 5 is given as below:

**Fig. 5:**
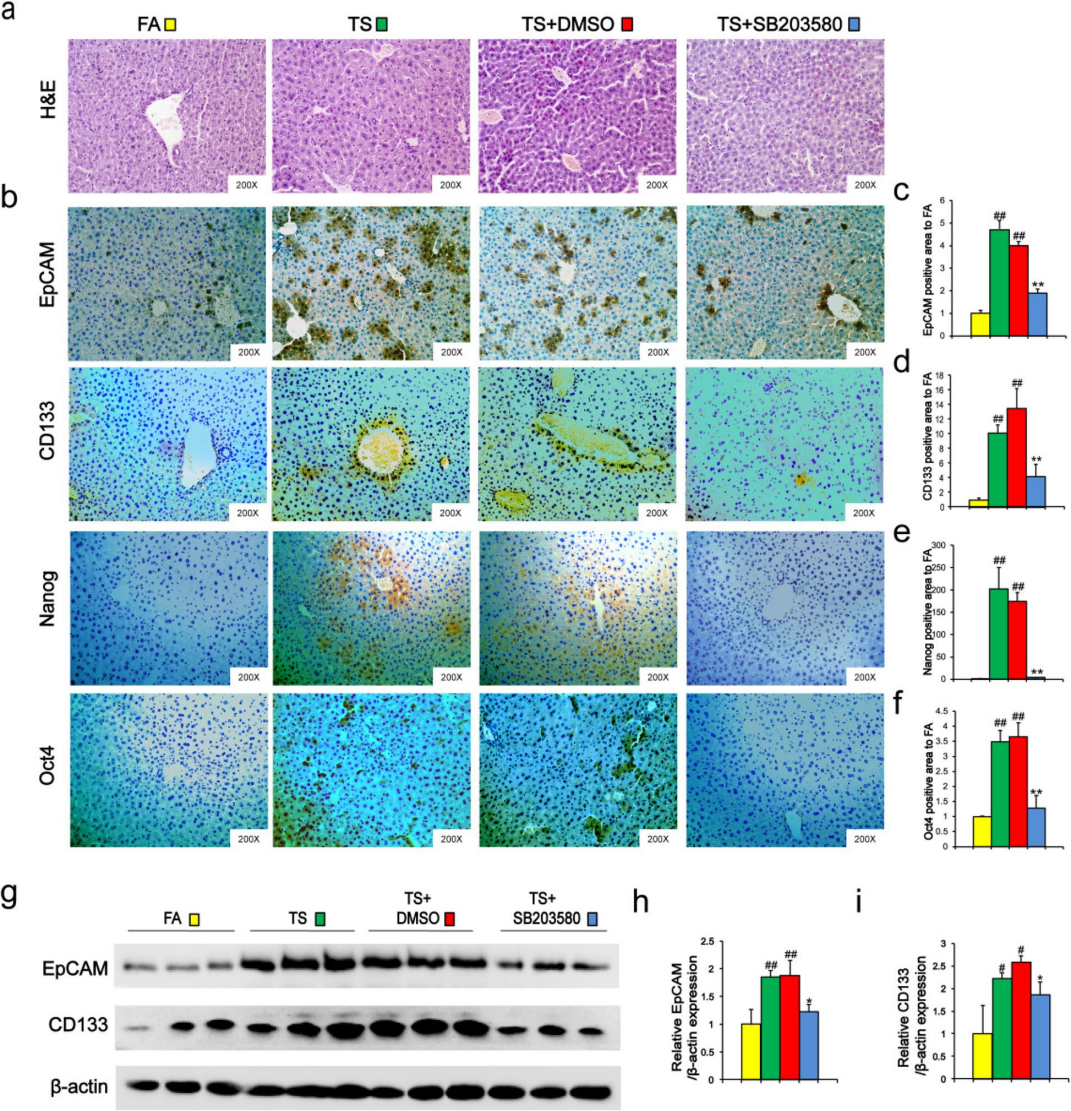
p38 suppression reversed long term TS exposure-triggered CSC-like properties. Mice exposed to TS were treated with or without p38 MAPK inhibitor (SB 203580) for 12 weeks, and representative micrographs of liver tissue were stained with H&E (**a**). **b** Immunohistochemical staining for EpCAM, CD133 and Nanog, Oct4 in liver tissues. **c-f** Fold changes of EpCAM (**c**), CD133 (**d**), Nanog (**e**) and Oct4 (**f**)—positive area in TS group compared with FA group. **g** Western blotting of EpCAM and CD133 in liver tissues. β-actin was served as the loading control. **h-i** The indicated proteins relative to β-actin were assessed by densitometric analysis; six animal samples per group were used for the densitometric analysis. Data are expressed as mean ± SD. The significance was assessed with one-way ANOVA test. # *P* < 0.05, ## *P* < 0.01, compared with FA control; * *P* < 0.05, ** *P* < 0.01, compared with TS + DMSO group. FA = filtered air; TS = tobacco smoke

The correction does not affect the overall results or conclusion of the article.
